# Administration of the Resveratrol Analogues Isorhapontigenin and Heyneanol-A Protects Mice Hematopoietic Cells against Irradiation Injuries

**DOI:** 10.1155/2014/282657

**Published:** 2014-06-24

**Authors:** Hui Wang, Yi-ling Yang, Heng Zhang, Hao Yan, Xiao-jing Wu, Chun-ze Zhang

**Affiliations:** ^1^Department of Radiation Oncology, Tianjin Union Medical Center, Tianjin 300121, China; ^2^Department of Breast Cancer Pathology and Research Laboratory, Key Laboratory of Breast Cancer Prevention and Therapy (Ministry of Education), Key Laboratory of Cancer Prevention and Therapy (Tianjin), National Clinical Research Center for Cancer, Tianjin Medical University Cancer Institute and Hospital, Tianjin 300060, China; ^3^Institute of Radiation Medicine, Peking Union Medical College (PUMC), Tianjin 300192, China; ^4^Department of Anorectal Surgery, Tianjin Union Medical Center, Tianjin 300121, China

## Abstract

Ionizing radiation (IR) is known not only to cause acute bone marrow (BM) suppression but also to lead to long-term residual hematopoietic injury. These effects have been attributed to IR inducing the generation of reactive oxygen species (ROS) in hematopoietic cells. In this study, we examined if isorhapontigenin and heyneanol-A, two analogues of resveratrol, could mitigate IR-induced BM suppression. The results of cell viability assays, clonogenic assays, and competitive repopulation assays revealed that treatment with these compounds could protect mice BM mononuclear cells (BMMNC), hematopoietic progenitor cells, and hematopoietic stem cells from IR-induced BM suppression. Moreover, the expression of genes related to the endogenous cellular antioxidant system in hematopoietic cells was analyzed. The expression and activity of SOD2 and GPX1 were found to be decreased in irradiated BMMNC, and the application of the resveratrol analogues could ameliorate this damage. Our results suggest that in comparison with resveratrol and isorhapontigenin, treatment with heyneanol-A can protect hematopoietic cells from IR-induced damage to a greater degree; the protective effects of these compounds are probably the result of their antioxidant properties.

## 1. Introduction

Radiation therapy is a common and effective tool in the management of a wide variety of tumors; in some cases, it may be the single best treatment for cancer. Bone marrow (BM) suppression is the most common dose-limiting side effect during radiation therapy [1, 2]; BM suppression is also the primary cause of death following accidental exposure of a patient to a high dose of total body irradiation. Myelosuppression, which can occur as a result of high total body irradiation, not only worsens the outcome of cancer treatment but also adversely affects the quality of life of cancer patients [2, 3]. However, the mechanisms by which ionizing radiation (IR) induces BM injury remain poorly understood, and no effective treatment has been developed to ameliorate this type of injury.

Injuries due to IR occur as a result of the ionization of water resulting in the formation of reactive oxygen species (ROS), notably hydroxyl radicals, increasing oxidative stress [4, 5]. Several studies have demonstrated that the induction of oxidative stress in hematopoietic cells is associated with sustained oxidative DNA damage; this results in a persistent loss of proliferative capacity in hematopoietic progenitor cells (HPCs) and hematopoietic stem cells (HSCs) [4, 6, 7]. Our recent studies have indicated that a persistent IR-induced increase in the production of reactive oxygen species (ROS) can be achieved in hematopoietic cells, in part, via the downregulation of superoxide dismutase (SOD) and glutathione peroxidase (GPX) and the upregulation of NADPH oxidase 4 (NOX4) [8, 9]. Several recent studies have also demonstrated that the induction of oxidative stress is primarily responsible for the loss of HSC self-renewal as well as the premature exhaustion of HSCs in mice that have mutations in the ATM [10] and deletion of FoxO3(s) [11]. These findings suggest that it may be possible to ameliorate IR-induced BM injury through treatment with a potent antioxidant.

Resveratrol (trans-3,5,4′-trihydroxystilbene, REV), a polyphenolic compound primarily found in grapes, is a potent antioxidant [12]. Accumulating reports have shown that REV can prevent or slow a wide variety of diseases related to oxidative stress, including cancer, cardiovascular diseases, and Alzheimer's disease [13]. It has been demonstrated that REV can act as a scavenger of hydroxyl, superoxide, and metal-induced radicals [12]. It is likely that the protective effects of REV against oxidative injury can be attributed to REV upregulating endogenous cellular antioxidant systems, such as SOD and GPX, rather than through directly scavenging ROS [9, 12]. Although the effects of REV in ameliorating IR-induced hematopoietic cell injuries have been investigated [9], little is known of the effects of oligomers of REV, such as isorhapontigenin (ISOR), a derivative of stilbene that can be isolated from* Belamcanda chinensis*, and heyneanol-A (HEY-A), a tetramer of REV that can be isolated from* Vitis heyneana*. Previous studies have revealed that these compounds possess anti-inflammatory, anti-apoptotic, and anti-oxidative activity [14, 15], but the biological activity of these compounds against irradiation injuries has not been investigated.

Owing to the remarkable therapeutic potential of REV's oligomers ISOR and HEY-A, we examined their effects on IR-induced BM suppression in our well-established and well-characterized mouse model. The results indicated that the two oligomers of REV could ameliorate IR-induced BM injury and that this occurred, at least partly, via the upregulation of the expression of SOD2 and GPX1 in hematopoietic cells.

## 2. Materials and Methods

### 2.1. Reagents

Anti-mouse-CD45.1-FITC (clone A20, Ly5.1), anti-mouse-CD45.2-PE (clone104, Ly5.2), anti-mouse-Ly6G/Gr-1-PE/Cy7 (cloneRB6-8C5), anti-mouse-CD45R/B220-PerCP (cloneRA3-6B2), anti-mouse-CD11b-PE/Cy7 (cloneM1/70), and anti-mouse-CD3-APC (clone145-2C11) antibodies were obtained from eBioscience (San Diego, CA, USA). REV and ISOR were purchased from Sigma (St. Louis, MO, USA). HEY-A was kindly provided by Dr. Qi Hou from the Institute of Materia Medica at Peking Union Medical College (PUMC, Beijing, China).

### 2.2. Mice

Male C57BL/6 mice were purchased from the Institute of Laboratory Animal Sciences (PUMC, Beijing, China) and were bred at the certified animal care facility in the Institute of Radiation Medicine of PUMC. All of the mice used in the study were aged approximately 8–10 weeks. The Institutional Animal Care and Use Committee of PUMC approved all the experimental procedures used in this study.

### 2.3. Treatment of IR-Exposed BM Mononuclear Cells with REV, ISOR, and HEY-A

The mice were euthanized using CO_2_; immediately following this, the femora and the tibiae were harvested from the mice. BM mononuclear cells (BMMNC) were isolated from the mice according to a previously described method [9, 17]; the BMMNC were incubated (1 × 10^6^/mL in complete medium) with REV, ISOR, HEY-A (0.01–100 *μ*M), or 0.2% dimethyl sulfoxide (DMSO; used as a vehicle control) at 37°C for 60 min. The cells were then exposed to 1, 2, or 4 Gy IR generated in an Exposure Instrument Cammacell-40 ^137^Cesium irradiator (Atomic Energy, Lin, CA) at a rate of 0.76 Gy/min and sham-irradiation cells were set. Cells were incubated at 37°C, 5% CO_2_, and 100% humidity for various durations, as indicated in the individual experiments.

### 2.4. Cell Viability Assays

The cells were plated into a 96-well plate (1 × 10^5^ cells/well in 100 *μ*L of medium) and were cultured for 18 h. Cell viability was monitored using the luminescent-based CellTiter-Glo system (Promega Corporation, Madison, WI, USA) according to the manufacturer's recommended protocols [16]. The luminescence of each well was read using an Infinite M200 multimode microplate reader (TECAN, Switzerland). Cell viability was normalized and expressed as a percentage of the untreated cells [17].

### 2.5. Colony-Forming Cells Assay

The colony-forming cells (CFC) assay was performed by culturing BMMNC in MethoCult M3534 methylcellulose medium (StemCell Technologies, Vancouver, BC, Canada) according to the manufacturer's instructions. BMMNC, incubated with REV, ISOR, or HEY-A (0.01–1 *μ*M), and irradiated as described above, were suspended in MethoCult M3534 medium at 2 × 10^4^ or 1 × 10^5^ viable cells/mL; the cells were then seeded in the wells of 24-well plates. The plates were incubated for 7 days. Colonies of ≥50 cells were scored under an inverted microscope [2, 17] and the results were expressed as the number of CFU-GM per 10^5^ cells.

### 2.6. Competitive Repopulation Assays

**Figure 1:**
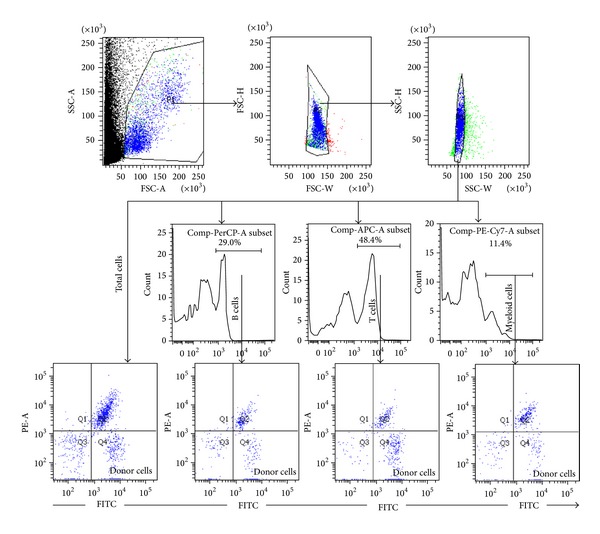
A representative gating strategy of the multilineage cell analysis performed by flow cytometry. After the red blood cells had been lysed in 0.15 M NH_4_Cl solution, the blood samples were stained with FITC-conjugated anti-CD45.1, PE-conjugated anti-CD45.2, PerCP-conjugated anti-B220, APC-conjugated anti-CD3, and PE/Cy7-conjugated anti-Gr-1 and CD11b. The samples were then analyzed using an LSR II flow cytometer.

Competitive repopulation assays were performed using the Ly5 congenic mouse system according to a previously described method [8, 9, 17]. After incubation with REV, ISOR, and HEY-A (1 *μ*M) or exposure to irradiation (2 Gy) as described above, the donor cells (C57BL/6-Ly-5.1 mice, 1 × 10^5^ BMMNC) were mixed with 1 × 10^5^ competitive BMMNC that was pooled from three Ly5.1/Ly5.2 hybrid mice. The cells were then transplanted by lateral canthus-vein injection into C57BL/6-Ly-5.2 mice (seven recipients/groups) that had received a lethal IR dose (9.0 Gy total body irradiation). To analyze the engraftment, peripheral blood was collected 2 months after transplantation, using heparin-coated micropipettes (Drummond Scientific, Broomall, PA, USA), from the medial canthus of all the recipients. Following this, the red blood cells were lysed in 0.15 M NH_4_Cl solution and the blood samples were stained using FITC-conjugated anti-CD45.1, PE-conjugated anti-CD45.2, PerCP-conjugated anti-B220, APC-conjugated anti-CD3, PE/Cy7-conjugated Anti-Gr-1, and CD11b antibodies and were analyzed by an LSR II flow cytometer (BD Bioscience, San Jose, CA, USA), as illustrated in Figure 1.

### 2.7. Quantitative Real-Time PCR Assays

BMMNC were incubated with REV, ISOR, and HEY-A or exposed to irradiation (2 Gy) as described above and the cells were incubated for 24 h. Total RNA was extracted from the BMMNC using TRIzol reagent (ABI Co., USA) following the manufacturer's protocol. First-strand cDNA was synthesized from total RNA using an RNA PCR Kit (AWV) Ver3.0 (TAKARA Co., Japan) according to the manufacturer's protocol. PCR primers for the SOD2, GPX1, and the housekeeping gene GAPDH were obtained from Sangon Biotech (Shanghai, China). The sequences of the primers used in this study were: SOD2, 5′-ATT AAC GCG CAG ATC ATG CA-3′ (forward) and 5′-TGT CCC CCA CCA TTG AAC TT-3′ (reverse); GPX1, 5′-TGC TCA TTG AGA ATG TCG CGT CTC-3′ (forward) and 5′-AGG CAT TCC GCA GGA AGG TAA AGA-3′ (reverse); GAPDH, 5′-TGA AGG TCG GTG TGA ACG GAT TTG GC-3′ (forward); and 5′-CAT GTA GGC CAT GAG GTC CAC CAC-3′ (reverse) [9]. cDNA samples were mixed with primers and SYBR Master Mix (ABI Co.) to a total volume of 25 *μ*L. All the samples were analyzed in triplicate using an ABI Prism 7500 Sequence Detection System (Applied Biosystems-Life Technologies). The thermal cycling conditions used in the protocol were 2 min at 50°C and 10 min at 95°C, followed with 40 cycles at 95°C for 15 s, and finally 60°C for 1 min. The threshold cycle (CT) values for each reaction were determined and the average CT value was calculated using TaqMan SDS analysis software (Applied Biosystems-Life Technologies). The changes in the level of expression of the target genes were calculated using the comparative CT method (fold changes = 2^[−ΔΔCT]^) as described previously [18].

### 2.8. Analysis of the Enzymatic Activity of SOD2 and GPX1

The enzymatic activities of SOD2 and GPX1 in BMMNC were analyzed using a SOD2 assay kit and a Cellular Glutathione Peroxidase 1 assay kit (Beyotime Institute of Biotechnology, Jiangsu, China); these assays were performed following the manufacturer's instruction, as described previously [9].

### 2.9. Statistical Analysis

The data were analyzed using an analysis of variance (ANOVA) test. In the event that the ANOVA test justified post hoc comparisons between the means of the group, these comparisons were made using the Student-Newman-Keuls test for multiple comparisons. Differences were considered significant at *P* < 0.05. The statistical analysis was performed using SPSS 16.0 software (SPSS Inc., Chicago, IL, USA).

## 3. Results

### 3.1. REV, ISOR, and HEY-A Protect BMMNC from Irradiation Injury* In Vitro*

**Figure 2:**
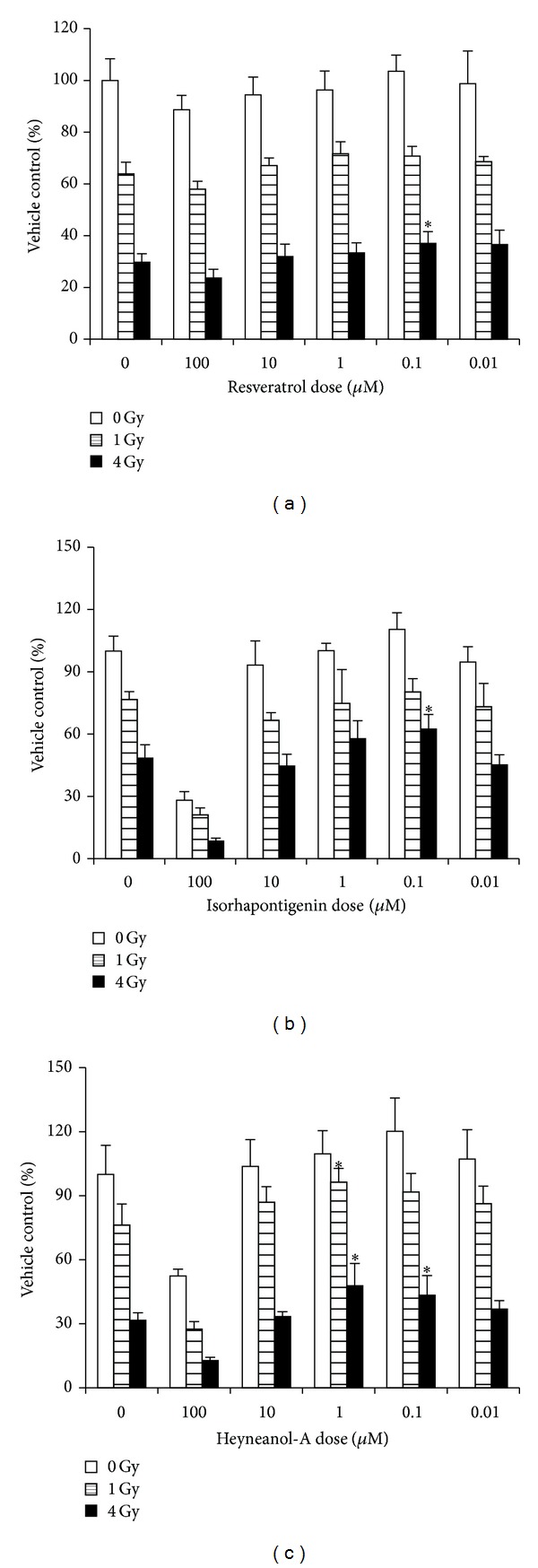
REV (a), ISOR (b), and HEY-A (c) reduce IR-induced suppression of the viability of BMMNC. Cells received treatment with control, REV, ISOR, or HEY-A before being sham-irradiated as a control or irradiated with 1–4 Gy IR; following this, the cells were cultured for 18 h. Cell viability was monitored as described in the paper text. Data are expressed as the relative mean viability ± SE. ∗*P* < 0.05 versus control, *n* = 6. REV: resveratrol; ISOR: isorhapontigenin; HEY-A: heyneanol-A; IR: ionizing radiation; BMMNC: bone marrow mononuclear cells.

Luminescence assays were performed to evaluate cell viability, as described in our previous work [17]. As shown in Figure 2, the viability of BMMNC decreased significantly after IR exposure. In comparison with the control group, the viability of irradiated (4 Gy) BMMNC increased by 24.6, 28.9, and 37.3–51.3% after being incubated with REV (0.1 *μ*M, *P* < 0.05), ISOR (0.1 *μ*M, *P* < 0.05), and HEY-A (0.01–0.1 *μ*M, *P* < 0.05), respectively. These data suggest that treatment with REV, ISOR, or HEY-A may be able to ameliorate IR-induced injuries in mice BMMNC and that, of the three, HEY-A has the most significant protective effect.

### 3.2. REV, ISOR, and HEY-A Increase the Ability of HPCs to Form Colonies of CFU-GM

**Figure 3:**
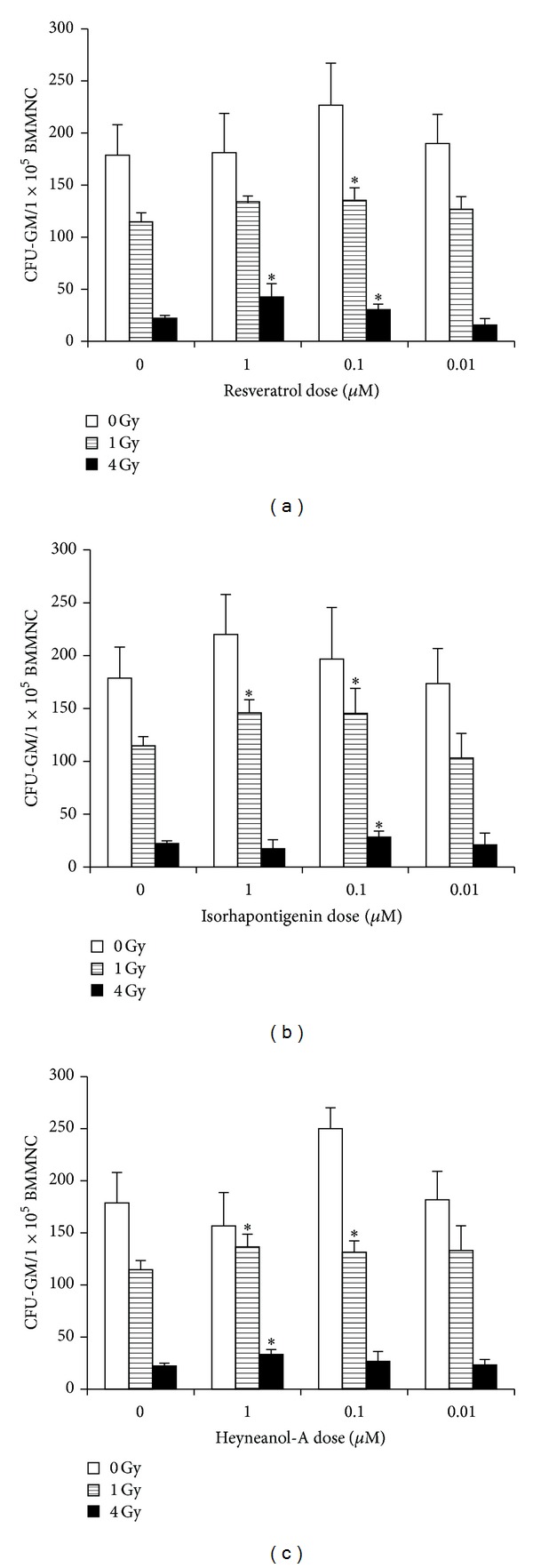
REV (a), ISOR (b), and HEY-A (c) reduce IR-induced suppression of HPC clonogenic function. Mice BMMNC received treatment with control, REV, ISOR, or HEY-A before being sham-irradiated as a control or sublethally irradiated with 1–4 Gy IR. The clonogenic function of HPCs and BMMNC was analyzed using a CFC assay. Colonies of ≥50 cells were scored under an inverted microscope on day 7 and the results are expressed as the number of CFU-GM per 10^5^ cells. Data are presented as the mean ± SE. ∗*P* < 0.05 versus control, *n* = 6. REV: resveratrol; ISOR: isorhapontigenin; HEY-A: heyneanol-A; IR: ionizing radiation; HPC: hematopoietic progenitor cell; BMMNC: bone marrow mononuclear cells; CFC: colony-forming cells.

The CFC assay was performed to evaluate the viability of HPCs affected by IR after treatment with REV, ISOR, and HEY-A [2, 8, 17]. The ability of BMMNC that had been treated with sham-irradiation or vehicles to form CFU-GM is shown in Figure 3. The cells that were exposed to different doses of IR (1–4 Gy) exhibited a diminished ability (35.8–87.5%) to form CFU-GM (*P* < 0.01), while treatment with the three compounds caused a moderate, but still significant, recovery in colony-forming abilities. In comparison with one of the IR groups (4 Gy), the number of colonies of CFU-GM increased by 91.5, 49.3 (*P* < 0.01), and 26.46% (*P* < 0.01) after treatment with REV (1 *μ*M), HEY-A (1 *μ*M), and ISOR (0.1 *μ*M), respectively. These results suggest REV, HEY-A, and ISOR could ameliorate IR-induced injuries in mice HPCs.

### 3.3. REV and HEY-A Enhance Long-Term and Multilineage Engraftment of Irradiated HSCs

**Figure 4:**
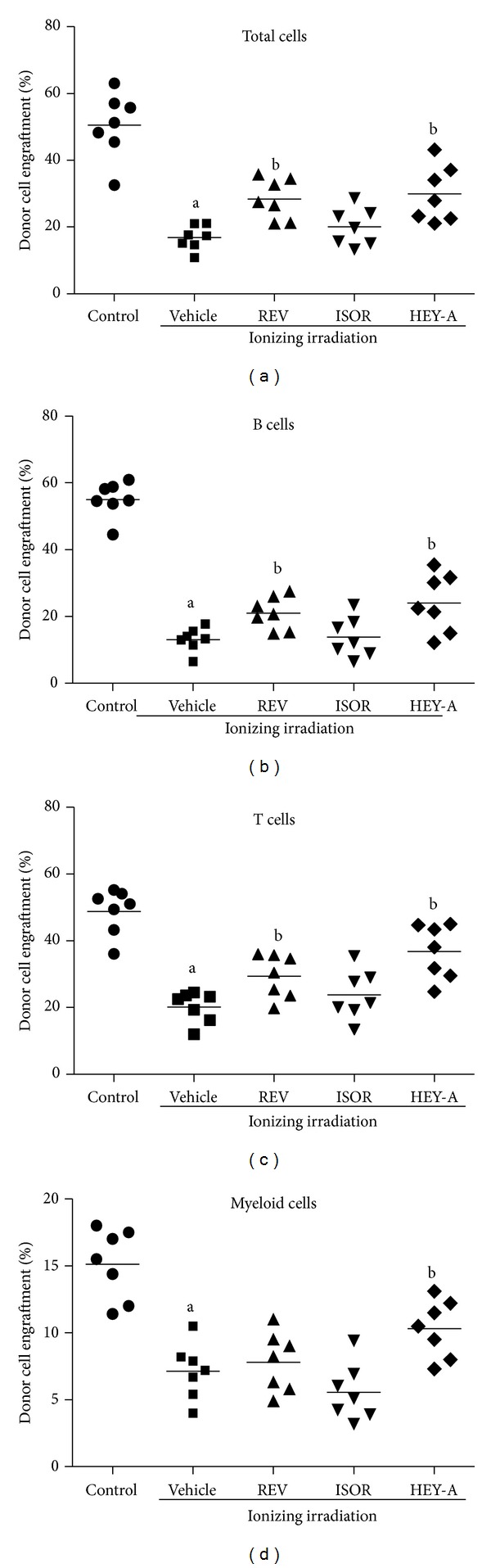
REV, ISOR, and HEY-A reduce IR-induced suppression of posttransplantation long-term engraftment of HSCs. Donor BMMNC received treatment with control, REV, ISOR, or HEY-A (1 *μ*M) before being treated with IR (2 Gy); the BMMNC were then mixed with competitive cells. Cells were transplanted into receptor mice as described in the paper text, and donor cell engraftment was analyzed 2 months after transplantation. The data are expressed as means ± SE of the percentage of donor-derived cells in the peripheral blood. (a) donor-derived leukocytes (CD45.1 + CD45.2 − cells), (b) donor-derived B cells (CD45.1 + CD45.2 − B220 + cells), (c) donor-derived T cells (CD45.1 + CD45.2 − CD3 + cells), and (d) donor-derived myeloid cells (CD45.1 + CD45.2 − CD11b + and/or Gr-1 + granulocyte-monocyte-macrophage). ^a^ *P* < 0.05 versus control; ^b^ *P* < 0.05 versus vehicle, (*n* = 7 recipient mice/group). REV: resveratrol; ISOR: isorhapontigenin; HEY-A: heyneanol-A; IR: ionizing radiation; HSC: hematopoietic stem cell; BMMNC: bone marrow mononuclear cells.

We performed long-term and multilineage engraftment assays, a gold standard in measuring HSC function, to validate whether treatment with these three compounds could ameliorate IR-induced functional declines in HSCs. As can be seen in Figure 4, at 2 months after transplantation, the mice that received donor cells that had been exposed to irradiation with vehicle treatment exhibited a substantial decrease in donor cell engraftment in all lineages. When treated with REV (1 *μ*M), the donor cell engraftment increased by 11.58% at 2 months, with increases of 7.86% in B cells, 9.16% in T cells, and 0.68% in myeloid cells derived from the donor cells. When treated with HEY-A (1 *μ*M), the donor cell engraftment increased by 13.07% at 2 months, with increases of 10.92% in B cells, 16.59% in T cells, and 3.17% in myeloid cells derived from donor cells. These findings suggest that treatment with REV and HEY-A can indeed preserve the functions of HSCs after IR exposure, resulting in enhanced long-term and multilineage engraftment after BM transplantation, in which HEY-A demonstrates a greater protective effect than REV.

### 3.4. REV, ISOR, and HEY-A Ameliorate IR-Induced Reductions in SOD2 and GPX1 Activity

**Figure 5:**
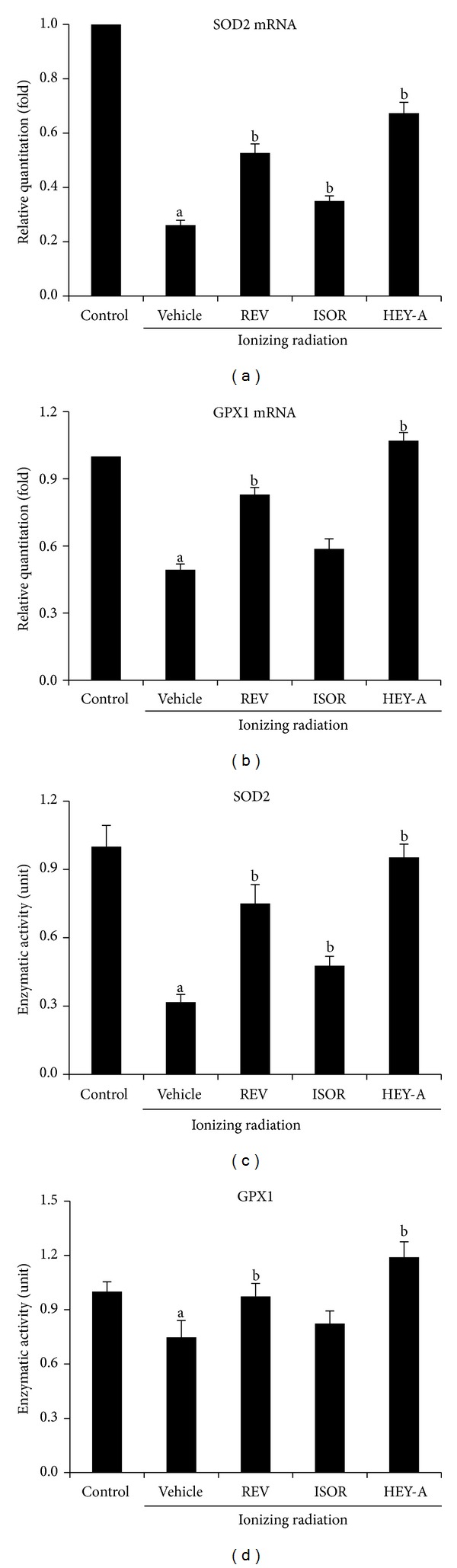
REV, ISOR, and HEY-A increase the enzymatic activity of SOD2 and GPX1 in BMMNC. The cells received treatment with control, REV, ISOR, or HEY-A (1 *μ*M) before being sham-irradiated as a control or irradiated with 2.0 Gy IR. The cells were incubated for 24 h before being analyzed. The expression of SOD2 and GPX1 mRNA was analyzed using qRT-PCR, and the enzymatic activities of SOD2 and GPX1 were analyzed using a SOD2 assay kit and a cellular GPX1 assay kit, respectively. ((a), (b)) Expression levels of SOD2 and GPX1 mRNA; ((c), (d)) enzymatic activity of SOD2 and GPX1 in BMMNC. Results of the mRNA-expression analysis and the enzymatic-activity analysis are expressed as means ± SE of changes in expression/activity in comparison with those of the control. ^a^ *P* < 0.01 versus control, ^b^ *P* < 0.01 versus vehicle, *n* = 3. REV: resveratrol; ISOR: isorhapontigenin; HEY-A: heyneanol-A; BMMNC: bone marrow mononuclear cells; IR: ionizing radiation.

In a recent study, we demonstrated that IR-induced ROS stress contributes to IR-induced BM failure in hematopoietic cells partly via downregulating the activity of the proteins SOD2 and GPX1 [9]. In this study, the expression of SOD2 and GPX1 in BMMNC that had been exposed to IR and treated with the therapeutic compounds was investigated. As indicated in Figures 5(a) and 5(b), IR exposure significantly downregulated the expression of SOD2 and GPX1. The expression of SOD2 and GPX1 decreased by 73.9 (*P* < 0.01) and 50.7% (*P* < 0.01), respectively, at 24 h. Following treatment with REV, ISO, and HEY-A, the expression of SOD2 was upregulated 1.02-, 0.35-, and 1.58-fold, respectively, and the expression of GPX1 was upregulated 0.68-, 0.19-, and 1.17-fold, respectively. The modulation of SOD2 and GPX1 expression by IR and the three compounds in BM cells was also confirmed by performing enzymatic assays, which are shown in Figures 5(c) and 5(d). These data suggest that radiation exposure could downregulate the expression of SOD2 and GPX1 in BMMNC and that REV's analogues, ISOR, and HEY-A could neutralize this IR-induced downregulation in SOD2 and GPX1 expression and activity.

## 4. Discussion

In this study, we examined whether the REV oligomers ISOR and HEY-A could inhibit IR-induced BM injury in mice. Our results demonstrate that treatment with HEY-A could protect hematopoietic cells from IR-induced injury much better than REV. Moreover, it was demonstrated that ISOR has a protective effect on BMMNC and HPCs, but not on HSCs, following IR-induced injury. The effects of REV and its oligomers on IR-induced BM injury are likely the result of their antioxidant properties.

REV and its oligomers are not conventional antioxidants that inhibit oxidative stress by scavenging free radicals directly. As demonstrated in our study, we found that REV and its oligomers may effectively upregulate the expression of SOD2 and GPX1 in hematopoietic cells. Consequently, the oligomers of REV may be more efficacious than other antioxidants that are commonly used as a medical countermeasure to IR-induced injury. In particular, considering that these compounds are natural products, inexpensive, and low in toxicity and that they have been used widely as food supplements, they may be very suitable for medical applications. However, at present, the results of this study do not allow us to determine whether these compounds function as a radiation protectant, a radiation mitigator, or both. Further studies, where cells are treated either before or after IR exposure, will be needed to clarify this.

Furthermore, the mechanisms by which REV and its oligomers differentially regulate the expression of SOD2 and GPX1 in hematopoietic cells have yet to be investigated. REV is a putative activator of SIRT1, a NAD(+)-dependent histone deacetylase that can regulate gene expression by modulating the structure of chromatin [19]. In addition, multiple nonhistone targets have also been described for SIRT1, including some transcription factors or cofactors such as Forkhead box class O (FOXO) transcription factors, nuclear factor kB, and peroxisome proliferator-activated receptor-co-activator 1a [20, 21]. However, which of these mechanisms is involved in the regulation of GPX1 and SOD2 expression in hematopoietic cells has not yet been identified.

In addition, exposure to IR not only induces BM injury but also causes tissue damage including fibrosis, inflammation, and apoptosis [22–24]; IR also plays a major role in many side effects of radiotherapy in cancer patients. In addition to having an adverse effect on the quality of life of cancer patients, IR-induced injuries can also worsens the outcome of cancer treatment. It has been shown that oxidative stress is also an underlying cause of the types of tissue damage (fibrosis, inflammation, and apoptosis) that can arise from IR exposure. Therefore, it will be also interesting to examine whether REV and its oligomers have the potential to be useful as therapeutic agents for the treatment of other types of IR-induced tissue damage. Interestingly, it has been reported that a variety of cancer cells, including gastric, colorectal, lung, breast, prostate, esophageal, and thyroid carcinomas, can be inhibited by REV and its oligomers [25, 26]. Therefore, REV and its oligomers have the potential to increase the therapeutic efficacy of radiotherapy, not only by reducing tissue injury but also by inhibiting tumor growth.

## References

[1] Wang Y, Schulte BA, LaRue AC, Ogawa M, Zhou D (2006). Total body irradiation selectively induces murine hematopoietic stem cell senescence. *Blood*.

[2] Meng A, Wang Y, Van Zant G, Zhou D (2003). Ionizing radiation and busulfan induce premature senescence in murine bone marrow hematopoietic cells. *Cancer Research*.

[3] Dainiak N (2002). Hematologic consequences of exposure to ionizing radiation. *Experimental Hematology*.

[4] Kam WW, Banati RB (2013). Effects of ionizing radiation on mitochondria. *Free Radical Biology & Medicine*.

[5] Puthran SS, Sudha K, Rao GM, Shetty BV (2009). Oxidative stress and low dose ionizing radiation. *Indian Journal of Physiology and Pharmacology*.

[6] Shao L, Lou Y, Zhou D (2013). Hematopoietic stem cell injury induced by ionizing radiation. *Antioxidants & Redox Signaling*.

[7] Li H, Wang Y, Pazhanisamy SK (2011). Mn(III) meso-tetrakis-(N-ethylpyridinium-2-yl) porphyrin mitigates total body irradiation-induced long-term bone marrow suppression. *Free Radical Biology and Medicine*.

[8] Wang Y, Liu L, Pazhanisamy SK, Li H, Meng A, Zhou D (2010). Total body irradiation causes residual bone marrow injury by induction of persistent oxidative stress in murine hematopoietic stem cells. *Free Radical Biology and Medicine*.

[9] Zhang H, Zhai Z, Wang Y (2013). Resveratrol ameliorates ionizing irradiation-induced long-term hematopoietic stem cell injury in mice. *Free Radical Biology and Medicine*.

[10] Ito K, Hirao A, Arai F (2004). Regulation of oxidative stress by ATM is required for self-renewal of haematopoietic stem cells. *Nature*.

[11] Miyamoto K, Araki KY, Naka K (2007). Foxo3a is essential for maintenance of the hematopoietic stem cell pool. *Cell Stem Cell*.

[12] Pervaiz S, Holme AL (2009). Resveratrol: its biologic targets and functional activity. *Antioxidants & Redox Signaling*.

[13] Baur JA, Sinclair DA (2006). Therapeutic potential of resveratrol: the *in vivo* evidence. *Nature Reviews Drug Discovery*.

[14] Li H, Wang A, Huang Y (2005). Isorhapontigenin, a new resveratrol analog, attenuates cardiac hypertrophy via blocking signaling transduction pathways. *Free Radical Biology and Medicine*.

[15] Jang MH, Piao XL, Kim HY (2007). Resveratrol oligomers from Vitis amurensis attenuate *β*-amyloid-induced oxidative stress in PC12 cells. *Biological and Pharmaceutical Bulletin*.

[16] Noah JW, Severson W, Noah DL, Rasmussen L, White EL, Jonsson CB (2007). A cell-based luminescence assay is effective for high-throughput screening of potential influenza antivirals. *Antiviral Research*.

[17] Zhang H, Wang YA, Meng A (2013). Inhibiting TGF*β*1 has a protective effect on mouse bone marrow suppression following ionizing radiation exposure in vitro. *Journal of Radiation Research*.

[18] Zhang H, Li J, Wang Y (2012). Retinoblastoma 94 enhances radiation treatment of esophageal squamous cell carcinoma in vitro and in vivo. *Journal of Radiation Research*.

[19] Lambeth JD (2007). Nox enzymes, ROS, and chronic disease: an example of antagonistic pleiotropy. *Free Radical Biology and Medicine*.

[20] Landry J, Sutton A, Tafrov ST (2000). The silencing protein SIR2 and its homologs are NAD-dependent protein deacetylases. *Proceedings of the National Academy of Sciences of the United States of America*.

[21] Napper AD, Hixon J, McDonagh T (2005). Discovery of indoles as potent and selective inhibitors of the deacetylase SIRT1. *Journal of Medicinal Chemistry*.

[22] Kitada M, Kume S, Imaizumi N, Koya D (2011). Resveratrol improves oxidative stress and protects against diabetic nephropathy through normalization of Mn-SOD dysfunction in AMPK/SIRT1- independent pathway. *Diabetes*.

[23] Carsten RE, Bachand AM, Baileya SM, Ullrich RL (2008). Resveratrol reduces radiation-induced chromosome aberration frequencies in mouse bone marrow cells. *Radiation Research*.

[24] Yang Y, Paik JH, Cho D, Cho J, Kim C (2008). Resveratrol induces the suppression of tumor-derived CD4^+^CD25^+^ regulatory T cells. *International Immunopharmacology*.

[25] Athar M, Back JH, Tang X (2007). Resveratrol: a review of preclinical studies for human cancer prevention. *Toxicology and Applied Pharmacology*.

[26] Lee EO, Lee HJ, Hwang HS (2006). Potent inhibition of Lewis lung cancer growth by heyneanol A from the roots of Vitis amurensis through apoptotic and anti-angiogenic activities. *Carcinogenesis*.

